# Bacterial Cell Surface Heterogeneity: A Pathogen's Disguise

**DOI:** 10.1371/journal.ppat.1002821

**Published:** 2012-08-30

**Authors:** Henny C. van der Mei, Henk J. Busscher

**Affiliations:** Department of Biomedical Engineering, W. J. Kolff Institute, University Medical Center and University of Groningen, Groningen, The Netherlands; The University of North Carolina at Chapel Hill, United States of America

## Why Is It Advantageous for Microorganisms to be Able to Disguise Themselves?

All interactions of microorganisms with their environment are surface phenomena, and therewith involve the properties of the microbial cell surface [1] and its possible disguise or hidden identity by an altered appearance. Since appearance is what one initially sees upon first encounter, a disguise always refers to surface properties, like cloths for people and hydrophobicity or charge for microorganisms.

Antimicrobials, for instance, first have to approach an organism and interact with its cell surface before they can become effective. Hydrophobic lactobacilli with a mean water contact angle of 66 degrees were found to be susceptible to nonoxynol-9 (a non-ionic spermicide) and vancomycin, whereas hydrophilic strains with a mean water contact angle of 32 degrees were resistant [2]. Analogously, cationic polyquaternium-1 was only effective against more negatively charged *Pseudomonas aeruginosa* strains with an isoelectric-point (pH where the bacterial zeta potential is zero) ranging from 1.3 to 1.9, whereas more positively charged strains with an isoelectric-point between 4.0 and 5.5 were resistant [3]. Also Nagant et al. [4] noticed that more negatively charged *P. aeruginosa* strains were more sensitive to a cationic antimicrobial, inhibiting biofilm formation.

These examples show that if a microorganism, or part of the population it belongs to, is able to change surface properties, this will allow the organisms to evade environmental attacks. Moreover, since adhesion to substratum surfaces depends on the properties of the interacting surfaces [5], the ability of an organism to produce clones with different surface properties will allow a strain to adhere to different surfaces, which may be considered a survival mechanism [6]. Clearly, these are beneficial traits for pathogenic organisms.

## How Can We Measure the Surface Properties of Individual Microorganisms or Subpopulations in an Axenic Culture?

In microbiology we like to believe that when we grow an axenic culture, all organisms are identical. This belief is wrong and stems from the fact that measurement of properties of an individual organism or subpopulation of clones is generally impossible, either by lack of a suitable technique or due to statistical limitations. Microscopic analysis of axenic cultures of lactobacilli has shown that part of a population can possess an electron dense, ruthenium red-uranyl acetate stained surface layer, but microscopic analysis can inevitably only comprise of small fraction of the number of organisms cultured [7]. Also atomic force microscopy [8], enabling measurement of bacterial cell surface adhesiveness at the level of an individual organism, suffers from the inability to quantify differences in adhesiveness between organisms in a statistically reliable manner. Fluorescence microscopy and flow cytometry are also used to quantify heterogeneity in bacterial suspensions, but have as a disadvantage that bacteria either need to be labeled with a fluorescent probe or have to be genetically modified in order to insert a fluorescent reporter gene. Using fluorescent reporter genes, Baty et al. [9], for instance, demonstrated that subpopulations of the marine bacterium *Pseudoalteromonas* sp. S91 switched on metabolic genes triggered by chitin-coated surfaces.

Particulate microelectrophoresis is possibly the only technique able to reliably quantitate cell surface heterogeneity in axenic cultures without prior cell labeling. In particulate microelectrophoresis, microorganisms are suspended in a liquid phase. A flow chamber is subsequently filled with this suspension, and a voltage between 75 and 150 V is applied over the chamber [10]. Negatively charged microorganisms are then attracted to the positive electrode, and positively charged organisms are attracted to the negative electrode. The velocity at which an organism travels is a direct measure of its electrophoretic mobility (or zeta potential). The use of image analysis subsequently enables measurement of the velocity of individual organisms, and depending on the measuring time, several hundreds of individual clones in an axenic culture can be monitored and quantitated with good statistical reliability. For instance, using particulate microelectrophoresis, 11 out of 12 fresh clinical isolates of Gram-negative *Porphyromonas gingivalis*, *Prevotella intermedia*, and *Actinobacillus actinomycetemcomitans* and of Gram-positive *Peptostreptococcus micros* (all periodontal pathogens) displayed heterogeneous populations with respect to pH-dependent electrophoretic mobilities [11]. For the Gram-negative strains, the more negatively charged subpopulation was in the majority, while the *P. micros* strains appeared to be composed mainly of a less negatively charged subpopulation.

It may sound surprising, but also the measurement of cell surface hydrophobicity using MATH (Microbial Adhesion To Hydrocarbons) as introduced by Rosenberg et al. [12], allows us to distinguish microbial subpopulations with different ability to adhere to the hydrocarbon phase, although not with the same straightforward interpretation as in particulate microelectrophoresis. This requires use of MATH in its so-called kinetic mode [13], where a microbial suspension is vortexed for different periods of time with a hydrocarbon phase and the optical density of the aqueous phase is measured as a function of the vortexing time. Initial removal of organisms by the hydrocarbon phase is taken as a measure of cell surface hydrophobicity. Interestingly, whereas for some strains, all organisms in the aqueous suspension finally adhere to the hydrocarbon phase after prolonged vortexing indicative of the absence of subpopulations with different cell surface hydrophobicities, for other strains, a sizeable fraction of all suspended organisms remains in suspension, indicative of a subpopulation with lower cell surface hydrophobicity.

## Is There Evidence That Cell Surface Heterogeneity Is a Trait of Pathogens and Do Other Strains Exhibit the Same Behavior?

Table 1 summarizes different strains and species for which cell surface heterogeneity in axenic cultures has been found. As can be seen, most evidence stems from particulate microelectrophoresis. Cell surface heterogeneity has been described mostly for pathogenic organisms. Surface heterogeneity can provide a part of a bacterial population with stealth-like properties, allowing at least a number of organisms to escape killing by antimicrobials, which enhances the pathogenicity of the population. Furthermore, since the properties of a microbial cell surface determine the organism's ability to adhere to a surface, the possession of heterogeneous cell surface properties allows organisms to adhere to a greater variety of surfaces. For *T. denticola* and *E. faecalis*, this has been demonstrated to be a clear pathogenic trait, as it allows the organism to adhere with greater versatility to its target substrata. However, also for non-pathogens like lactobacilli, the ability to adhere to a wide range of different surfaces offers an advantage, as adhesion very often is a survival mechanism, stimulating the organisms to adapt a protective, biofilm mode of growth.

**Table 1:** Summary of microbial strains for which clonal subpopulations expressing phenotypes with different cell surface properties have been found.

Microbial Strain	Technique	Result	Reference
Lactobacilli	Particulate microelectrophoressis	- Serial passaging result in increased proportions of bacteria with a thicker cell wall and more negative zeta potential.	[7]
Periodontal pathogens	Particulate microelectrophoresis	- Majority populations in axenic cultures of Gram-negative bacteria are highly negatively charged.- Most negatively charged subpopulation of *Treponema denticola* in an axenic culture adheres to erythrocytes.	[11], [18]
*Enterococcus faecalis*	Particulate microelectrophoresis and flow cytometry	- Clinical isolates and laboratory strains display heterogeneous surface charge.- Surface heterogeneity is not caused by quorum sensing, not plasmid mediated, and independent of *esp* and Agg.- Culture heterogeneity enhances adhesion to abiotic surfaces.- Less negatively charged subpopulations have pili, mediating adhesion to platelets, fibrinogen, and collagen and thus are more virulent.	
*P. aeruginosa*	Particulate microelectrophoresis	- Most negatively charged subpopulations are more sensitive to cationic antimicrobials.	[3], [4]
*Staphylococcus epidermidis*	Particulate microelectrophoresis and congo red agar plating	- Heterogeneous cultures with respect to surface charge and slime production form more extensive biofilms.	[14]
*Listeria monocytogenes*	Atomic force microscopy	- Bacterial virulence is higher for cultures showing greater variability in adhesion forces.	[20]
*Vibrio cholerae*	Fluorescence microscopy and flow cytometry	- Bacteria expressing *tcpA* in heterogeneous cultures are more virulent.	[16]

## How Do Bacteria Regulate Cell Surface Heterogeneity?

**Figure 1:**
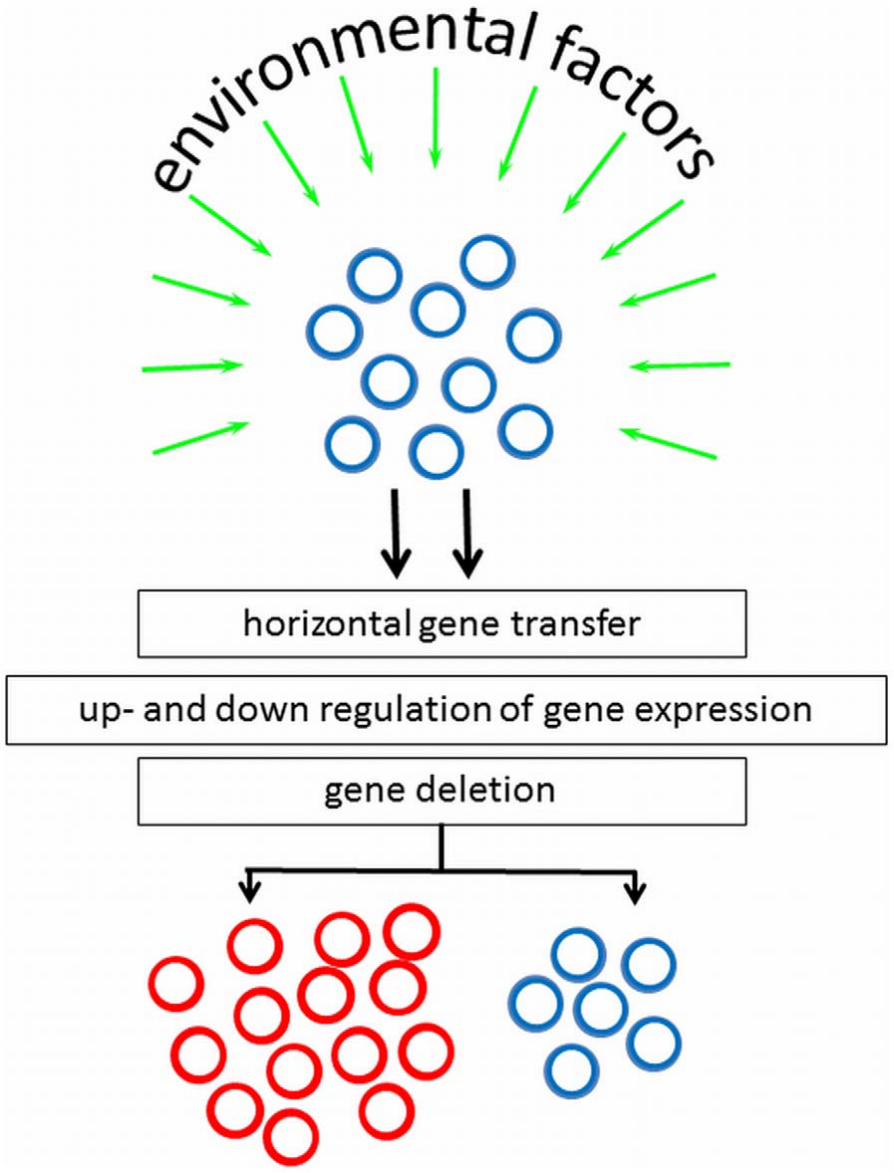
Environmental factors and genotypic mechanisms causative to appearance of subpopulations with altered phenotypic appearance, starting from a culture with homogeneous cell surface properties. Note that inverse processes can also be envisaged.

Bacteria can adapt quickly to a new environment triggered by environmental signals to change their phenotypic appearance, but it is of apparent advantage that not all clones in a population do so. The genotypic mechanisms and environmental factors controlling surface heterogeneity in axenic cultures are only recently being studied and no general mechanism can yet be forwarded. However, pathogens migrating through the human body encounter different micro-environments, and in response to their environment, virulence genes could be horizontally transferred, up- or down-regulated, or deleted (see Figure 1). Although cell surface heterogeneity was observed in 5% of clinical *S. epidermidis* isolates [14], it may not be ruled out that in vitro culturing, including medium selection and serial passaging, influences the occurrence of bacterial cell surface heterogeneity.

Bicarbonate may play a determinant role in the development of culture heterogeneity. Bicarbonate as produced by mammalian cells is known to enhance the production of virulence factors in, for example, *V. cholera*, *Staphylococcus aureus*, *Bacillus anthracis*, while in *E. faecalis* bicarbonate increases pilus formation regulating its colonization of surfaces [15]. In *V. cholera*, the genes encoding the toxin-co-regulated pilus (TCP) and the cholera toxin (CT) are up-regulated by the excretion of bicarbonate by epithelial cells early in the infection process, causing increased adhesion to these epithelial cells. Significant heterogeneity was subsequently observed late in the infectious process, with a TCP/CT expressing and TCP/CT non-expressing subpopulation [16], because bacteria more distant from the epithelial cells did not receive the necessary signals from the epithelial cells. Bacterial infections are often caused by bacteria adhering to tissues and biomaterial-implants in a biofilm-mode of growth. In a biofilm, organism are comprised in different micro-environments with respect to nutrient availability, oxygenation, osmolarity, and cell density [17], which may all constitute environmental stimuli for phenotypic changes.

## What Are the Implications of Cell Surface Heterogeneity for Future Pathogen Control?

Development of new antimicrobials and strategies for pathogen control are usually based on evaluating efficacy at the level of entire populations, discarding the possible existence of heterogeneous subpopulations. We have shown that axenic bacterial cultures in vitro, as well populations of infecting pathogens in vivo, can display heterogeneous surface properties, which puts them at an advantage in comparison with bacterial populations possessing similar phenotypic properties across an entire population. These advantages either include the ability to exert a stronger virulence towards the host or increased possibilities to adhere and survive antimicrobial and other environmental attacks. This implies that in the development of new antimicrobials and strategies for pathogen control, it is important to account for surface heterogeneity, as a disguised subpopulation may form the basis for surviving clones to form more virulent and antimicrobial-resistant strains.
